# Thin-Film Transistor-Based Biosensors for Determining Stoichiometry of Biochemical Reactions

**DOI:** 10.1371/journal.pone.0169094

**Published:** 2016-12-29

**Authors:** Yi-Wen Wang, Ting-Yang Chen, Tsung-Han Yang, Cheng-Chung Chang, Tsung-Lin Yang, Yu-Hwa Lo, Jian-Jang Huang

**Affiliations:** 1 Graduate Institute of Photonics and Optoelectronics, National Taiwan University, Taipei, Taiwan; 2 Graduate Institute of Biomedical Engineering, National Chung Hsing University, Taichung, Taiwan; 3 Department of Otolaryngology, National Taiwan University Hospital and College of Medicine, National Taiwan University, Taipei, Taiwan; 4 Research Center for Developmental Biology and Regenerative Medicine, National Taiwan UniversityTaipei, Taiwan; 5 Materials Science and Engineering Program, University of California San Diego, La Jolla, California, United States of America; 6 Department of Electrical and Computer Engineering, University of California San Diego, La Jolla, California, United States of America; 7 Department of Electrical Engineering, National Taiwan University, Taipei, Taiwan; Institute of Materials Science, GERMANY

## Abstract

The enzyme kinetic in a biochemical reaction is critical to scientific research and drug discovery but can hardly be determined experimentally from enzyme assays. In this work, a charge-current transducer (a transistor) is proposed to evaluate the status of biochemical reaction by monitoring the electrical charge changes. Using the malate-aspartate shuttle as an example, a thin-film transistor (TFT)-based biosensor with an extended gold pad is demonstrated to detect the biochemical reaction between NADH and NAD^+^. The drain current change indicates the status of chemical equilibrium and stoichiometry.

## Introduction

A biochemical reaction is the process that transforms one molecule to another within a cell. It is often catalyzed by enzyme that alters the specificity and equilibrium of the reaction. Therefore, enzymatic kinetics is critical for detecting biochemical[1–3] and for understanding the catalytic mechanisms of the enzyme[4,5]. In addition to the catalytic reactants, the stoichiometric reactants are also important in a biochemical reaction. The stoichiometry can be employed to understand the efficiency of metabolic pathways. The biochemical reactions are usually measured by methods including gas monitoring [6], colorimetric assay [7] and pH value detection [8], which follow the changes of concentrations of the reagents or products. The importance of monitoring and determining the reaction status of the biochemical reaction is evidenced in scientific research and industrial applications.

The biochemical reaction can be categorized into a few types, including reduction and oxidation, water addition and removal, bond breaking reactions and the movement of groups between molecules. The complexity of life results from the above simple reactions occurring in various situations. Using the following chemical reaction as an example: reactions between NADH (Nicotinamide Adenine Dinucleotide, reduced form), MDH (Malate dehydrogenase) and OAA (Oxaloacetic Acid, the conjugate acid of oxaloacetate) are a part of the metabolic pathway in malate-aspartate shuttle. MDH catalyzes the reduction of oxaloacetate to malate using the oxidation of NADH to NAD^+^ [9]. In general, the reaction rate increases with the aid of MDH. A conventional way to detect the above reaction processes is by measuring the UV(ultraviolet) absorption of NADH at the wavelength of 340nm using a spectrophotometer, which differentiates the optical absorption between NAD^+^ and NADH [10]. Fluorescent properties are the alternative method to detect NADH and NAD^+^. NADH has an emission peak at the wavelength of 460nm and a decay time of 0.4 ns while NAD^+^ does not possess the emission at 460nm[11].

Optical methods to evaluate the bioreaction process based on the existence of NADH are straightforward. However, they may not be applicable to quantitatively determine reaction species and measure reaction coefficient under a reaction system at low reagent concentrations. Since most biomolecules carry electrical charges, we propose a charge-current transducer (a transistor) to monitor the biochemical reactive species so that reaction kinetics can be verified. The transistor-based biosensor provides the advantages of real-time sensing, simplicity, compactness, high-sensitivity, and cost-effectiveness. It is also suitable for on-chip integration with semiconductor integrated circuit technology [12]. In the literature, most FET (field effect transistor)-based biosensors were employed to study the concentration of the bio-solution [13–16] based on the amount of electrical charges with respect to the target molecules. In this work, instead of using a thin-film transistor (TFT) sensor for solely detecting molecular concentration, we aim at exploring the biochemical reaction status based on the electrical response. An IGZO (Indium Gallium Zinc Oxide) thin-film was employed as the transistor channel layer. As compared with organic TFTs, nanostructure FETs or transistors using 2D (two-dimensional) material as the channel layer, the IGZO TFTs have the advantages of mature fabrication processes, electrical and material stability and reliability. They can be patterned in array and fabricated on the glass substrates using sputtering technique, which makes low-cost mass production possible. Also, the IGZO TFT sensors can be immune to ambient light induced noise as the high bandgap IGZO has high optical transmission under visible light. Our results were further compared with the method based on optical spectrometry. The oxidation and reduction processes of NADH and OAA are an example. The proposed approach can be applied to similar bioreactions.

## Devices and Methods

### Biosensor device fabrication

**Fig 1:**
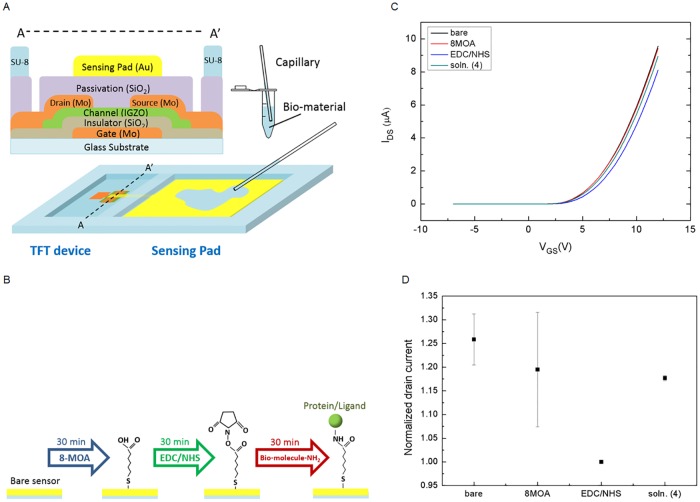
Structure of the TFT-based biosensor and the experimental flow. (A) Structure and cross-section of the TFT-based biosensor. (B) 8-MOA, EDC/NHS and target protein and ligand were applied to sensing pad step by step. (C) From (b), I_DS_-V_GS_ transfer curves of a bare TFT device along with the current response after the step-by-step application of 8MOA, EDC/NHS and soln. (4). (D) From (c), normalized drain currents at V_GS_ = 8V and V_DS_ = 5V can be derived. The drain current of after dispensing EDC/NHS is normalized to 1. The average current increment of soln. (4) is about 17.7% compared to that of EDC/NHS.

The sensor device is composed of a TFT (thin film transistor) to sense and read electrical signals and a dispensing pool for biomaterial solution, as shown in Fig 1A. The fabrication starts from TFT (thin film transistor) process and followed by the formation of the sensing pool. The gate contact metal molybdenum (Mo) was first deposited on the glass substrate by DC sputtering. The gate electrode was defined by photolithographic patterning and reactive-ion etching (RIE). SiO_2_ gate dielectric was deposited by plasma-enhanced chemical vapor deposition (PECVD). Next we sputtered and patterned the IGZO thin film channel layer by RF sputtering and Mo source/drain electrodes by DC sputtering. The TFT process was finalized by passivating the sample with a SiO_2_ layer to avoid channel layer oxidation, humidity and other physical and chemical damages. The biosensing region is realized by coating an extended gold sensing pad. Finally, the sensing pool is formed by photolithography using SU-8 photoresist, which separates the TFT and sensing pad. IGZO TFT arrays fabricated on the glass substrate have the advantages of stability, reliability and manufacturability, making the current work feasible for future commercial applications.

### Experimental flow for monitoring biochemical reaction

The experimental flow is illustrated in Fig 1B. The 8-MOA solution (8-mercaptooctanoic acid) with a concentration of 10mM was first applied on the gold sensing pad, following by 400mM EDC (ethyl(dimethylaminopropyl) carbodiimide)/100mM NHS (N-Hydroxysuccinimide) solution and bio-molecules with NH_2_ functional group. At each step, we waited for 30 minutes before washing to remove the unbonded molecules. For 8-MOA and EDC/NHS, the sensing pool was washed by deionized water while the bi-molecules with NH_2_ was by PBS (Phosphate-buffered saline). At this step, the sensor was considered as functionalized for target protein and ligand detection.

### Data extraction

The electrical signals of the transistors were characterized by Agilent 4155C semiconductor parameter analyzer. The transfer curves were measured at drain-source voltage, V_DS_, of 5V and gate-source voltage, V_GS_, sweeping from -7V to 12V. The drain current at V_GS_ = 8V was then extracted for further discussion.

### Optical spectrometry measurement

Various solutions were applied to a 96 well plate and absorptions at the wavelength of 340nm were detected by Spectra Max M Series Multi-Mode Microplate Readers for ELISA (enzyme-linked immunosorbent assay) measurement. Cutoff filters were used to reduce stray light and minimize background interference. A high-powered Xenon flash lamp was employed as the light source.

## Results and Discussion

The electrical charges carried by the biomaterials are transconducted and sensed by a TFT. The biomaterials were dispensed on the sensing pad, which acts as the top gate of the TFT **(see** Fig 1A**)**. Prior to the measurement, the sensor was functionalized by adding the cross linker, 8-MOA (8-mercaptooctanoic acid), EDC (ethyl(dimethylaminopropyl) carbodiimide)/NHS (N-Hydroxysuccinimide) solutions and bio-molecules with NH_2_ functional group as illustrated in Fig 1B. The current-voltage behaviors, I_DS_-V_GS_ transfer curves of the TFT, are shown in Fig 1C. The formation of 8MOA monolayer on the sensing pad causes the decrease of drain current, which further drops with the application of EDC/NHS solution to form a cross-linker layer. To understand the correlation between induced charges (drain current change) and the concentration of target solution, we designed five different solutions. In this experiment, PBS (Phosphate-buffered saline) buffer, 0.14mM NADH (denoted as soln. (1)), 0.14mM NADH + 1mg/ml OAA (soln. (2)), 0.14mM NADH + 0.36unit/ml MDH (soln. (3)) and 0.14mM NADH + 1mg/ml OAA + 0.36unit/ml MDH (soln. (4)) ^17^ were prepared. The solution of soln. (4) is designed to reach reaction equilibrium with the aid of MDH catalyst so that very small amount of NADH and OAA remains in the solution [17]. The nearly complete reaction will be verified by spectrophotometer later in this work. As we applied soln. (4) to the sensor for 30 minutes, a dramatic increase of the drain current, from that of the functionalized device, was observed. In Fig 1D, extracted from Fig 1C, the normalized drain current at V_GS_ = 8V and V_DS_ = 5V is shown, in which soln. (4) increases the drain current by 17.7%.

**Fig 2:**
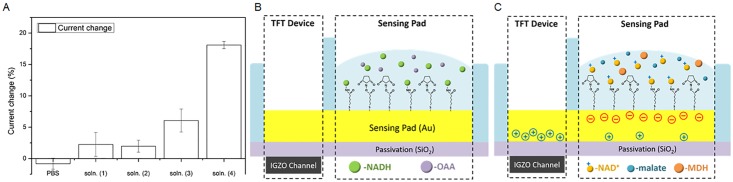
Percentage drain current changes at different experimental stages and illustration of the sensing mechanism. (A) Comparison of current changes of PBS, soln. (1), (2), (3) and (4). (B) Illustration of the incomplete reaction when only NADH and OAA are presented in the solution. Since no (or very small amount of) NAD+ is produced, the TFT device doesn’t sense significant amount of current changes. (C) For a nearly complete reaction when MDH catalyzes NADH and OAA to NAD^+^ and malate. NAD^+^ molecules carrying positive charges are captured by cross linker layer, contributing to the drain current increase. Negative charges are induced in the IGZO channel layer through the Au sensing pad separated by the passivation SiO_2_ from the channel.

The current changes are summarized in Fig 2A. The drain current change after adding PBS buffer is not significant. Soln. (1) and soln. (2) show a small amount of drain current changes by, 2.3% and 1.9%, respectively. The increase, despite small, is attributed to the binding of NADH molecules with the cross-linker layer. Furthermore, the drain current change of soln. (3), possibly caused by the interaction of NADH and MDH molecules on the linker, is about 6.1%. It is also likely that this current change, which is slightly larger than that of soln. (1) and soln. (2), is associated with the binding between NADH and MDH, and the interaction between cofactor and enzyme.

Among the solutions, the 17.7% drain current change of soln. (4) is the largest, which implies that the binding mechanism and the chemical reaction are different from the rest of the test samples. By examining the reaction:

$$NADH+H^{+}+oxaloacetate\overset{MDH}{\Leftrightarrow }NAD^{+}+malate$$ (1)

NAD^+^ plays a critical role in TFT current sensing as NADH was almost completely oxidized to NAD^+^ and OAA was almost completely reduced to malate with the aid with MDH for soln. (4). In such a case, NAD^+^ molecules were captured by the linkers, resulting in a large drain current change. The difference of current changes can thus be used to evaluate the reactivity and the status of reaction. For soln. (4) in Fig 2A, MDH catalyzes the redox reaction toward the products of the chemical eq (1), which speeds up the consumption of reactants. The large drain current change suggests the production of extra charged molecules in the solution.

The sensing mechanism is explained by visualizing the molecules on the gold sensing pad as shown in Fig 2B and 2C. In Fig 2B, when the solution with both NADH and OAA is dispensed, NADH molecules are captured by the cross linker layer without causing too much change on the drain current. While in Fig 2C, NAD^+^ molecules carrying positive charges are captured by cross linkers, inducing negative charges on gold sensing pad. Thus positive charges are induced at the bottom of the sensing pad, especially on the TFT side for electrical neutrality. The negative carriers induced in the IGZO channel contribute to the increment of the drain current.

**Fig 3:**
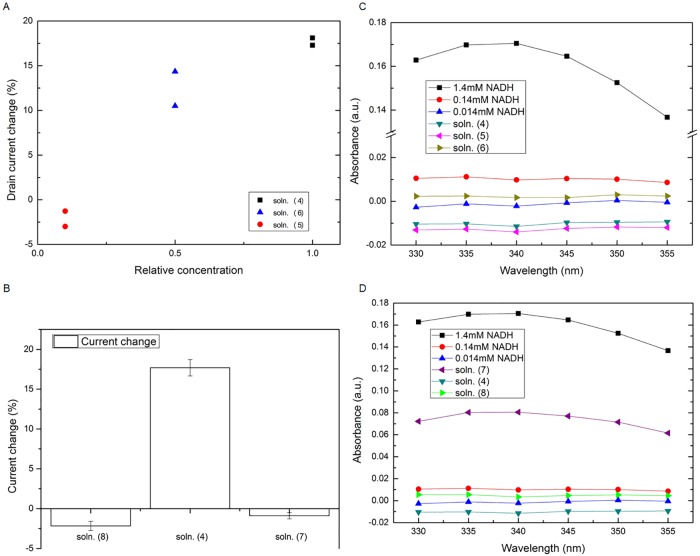
Experimental results: Comparisons of the corresponding drain current changes; and optical spectrometry measurement of various solutions. (A) For the nearly complete reaction, the drain current decreases when the concentration is reduced. (B) In comparison, for the incomplete reaction (soln. (7) and (8)), the tenfold increase or decrease of NADH concentration results in a small amount of drain current change. (C) Absorption spectra of pure NADH; along with solutions of nearly complete reaction for comparisons. (D) Absorption spectra of solutions of incomplete reaction are plotted for comparisons.

To further understand the relationship between current change and the concentration of reacted solutions, we prepared, in addition to soln. (4) described above, two other concentrations, 0.07mM NADH + 0.5mg/ml OAA + 0.18unit/ml MDH (denoted as soln. (5)), and 0.014mM NADH + 0.1mg/ml OAA + 0.036 unit/ml MDH (denoted as soln. (6)). As shown in Fig 3A, the drain current increase of soln. (4) is the largest, while that of soln. (5) (half the concentration of soln. (4)) is approximately 12.4% in average; and little variations, about -1.5% in average, for soln. (6). The above results indicate that forward reaction in eq (1) which leads to a large amount of NAD^+^ products can be detected by our device. The limit of detection, for the cases of nearly complete reaction, is in the range between the concentration of soln. (5) and (6) in the experiment.

Next, the reaction involving different concentrations of NADH was investigated. The concentrations of OAA and MDH remain unchanged with respect to soln. (4), while only that of NADH is varied. In Table 1, soln. (7) possesses 10 times the concentration of NADH with respect to soln. (4), while soln. (8) has only 1/10 of NADH. The corresponding drain current changes are summarized in Fig 3B. Interestingly, we observe very little change of the drain current for incomplete forward reacted soln. (7) and soln. (8). Only the forward and nearly complete reacted soln. (4) has the most obvious drain current response. When we review the chemical reaction, for soln. (7), the presence of NADH hinders the produced NAD^+^ molecules from being captured by cross-linker layer even though NAD^+^ molecules are produced during the reaction. On the other hand, when the concentration of NADH decreases 10 times for soln. (8), the small current change is correlated to the small amount of NAD^+^ molecules produced (same the result as soln. (6)). A summary of the above experiment is listed in Table 1.

**Table 1:** Summary of the TFT current changes of different solutions.

Soln.	NADH (mM)	OAA(mg/ml)	MDH(unit/ml)	Average current change (%)
(1)	0.14			2.3
(2)	0.14	1		1.9
(3)	0.14		0.36	6.1
(4)	0.14	1	0.36	17.7
(5)	0.07	0.5	0.18	12.4
(6)	0.014	0.1	0.036	-1.5
(7)	1.4	1	0.36	-2.1
(8)	0.014	1	0.36	-0.9

To benchmark our proposed method, the chemical reaction was examined by typical optical spectrometry. Since NADH has strong optical absorption at the wavelength of 340nm while no obvious absorption for NAD^+^ at that wavelength, an optical spectrometer was used to monitor the reaction. Fig 3C shows the absorption spectra of pure NADH solutions of three different concentrations. Soln. (4), (5) and (6) that reach steady-state reaction were also benchmarked. For pure 1.4mM NADH solution, the absorbance is much larger, more than ten times, than that of the rest two solutions, i.e. 0.14mM NADH and 0.014mM NADH solutions. The measurement suggests the detection limit of the spectrometer is above 0.14 mM NADH, as the 0.014mM NADH solution is out of the detection range. The steady-state reactions of soln. (4), soln. (5), and soln. (6) can be determined if no optical absorption is observed at 340nm when NADH is almost all reacted to the produced NAD^+^ molecules. Under such a condition, the absorptions of all three solutions (soln. (4), soln. (5), and soln. (6)) are very close to zero. Moreover, due to the interference of PBS buffer, soln. (4) and soln. (5) solutions show negative optical absorption by the spectrometer. For the above cases, we can only determine that no detectable amount of NADH was present with the concentration above 0.14mM. Hardly can we determine if the reaction is nearly complete or the concentration is below the limit of detection. Alternatively, our TFT sensor can indicate whether the reaction is complete even though no optical absorption is observed at 340nm. Our approach not only detects the produced NAD^+^ molecules, the electrical device also reflects the fact that NADH hinders charge sensing of NAD^+^ in the solution.

In addition, soln. (4), (7) and (8) were measured by the optical spectrometry. As shown in Fig 3D, for the case of soln. (7), the absorbance is about 0.08a.u. at the wavelength of 340nm, which is lower than that of pure 1.4mM NADH solution but much higher than that of other four solutions. The optical response of soln. (7) is around half of pure 1.4mM NADH, suggesting an incomplete reaction with around half of NADH remained in the solution. On the other hand, for soln. (8), only the information that very small amount of NADH is known from the optical response, hardly can we understand the biochemical status of complete or incomplete reaction.

As we compare the optical method to our proposed TFT-biosensor approach, our device can determine the stoichiometry of the biochemical reaction. The detection range is the concentration above soln. (5). Our approach has lower limit of detection compared to the existing optical method. It also rules out the problem of differentiating a chemical reaction with low concentration and a reaction with a significantly lower NADH solution.

## Conclusions

We propose a charge-current transducer (a transistor) to monitor the stoichiometry of biochemical reaction by observing the current changes from positive charges carried by NAD^+^ molecules in the biochemical reaction. The research focuses on the MDH-dependent reaction status. In the solution with nearly complete reaction, the current response becomes higher than that with reagents remained. We are able to understand the occurrence of the chemical reaction, verify the limit of detection, and differentiate the complete and incomplete reaction by the TFT current changes. The results were also compared with the optical spectrometry, which identifies the reaction by detecting the optical absorption of NADH. Our approach provides a better way to identify the reaction status, and meanwhile a higher sensitivity.
